# Irreducible Isolated Radial Head Dislocation in a Child Due to Annular Ligament Interposition: A Case Report

**DOI:** 10.7759/cureus.79694

**Published:** 2025-02-26

**Authors:** Luís Fabião, Vítor Macedo-Campos, Rita Ferreira de Castro, Miguel Lopes, Duarte Nuno Cadavez

**Affiliations:** 1 Orthopedics and Traumatology, Unidade Local de Saúde de Barcelos/Esposende, Barcelos, PRT

**Keywords:** annular ligament, irreducible radial head, pediatric elbow trauma, radial head dislocation, radial head open reduction

## Abstract

The annular ligament of the elbow is essential for its stability, playing a key role in both the proximal radioulnar and humeroradial joints, as well as supporting surrounding muscles and ligaments. Radial head dislocation is rare in children, and when isolated they can be challenging to reduce and may require surgical intervention.

An 11-year-old boy presented with an anteromedial dislocation of the radial head after a fall. Initial, closed reduction was attempted and failed, requiring surgical intervention, where we found a rupture of the annular ligament and interposition, which was repaired after reduction. At one year of follow-up, the patient achieved full range of motion and stability of the elbow.

Radial head dislocation is rare in children and even more rare without associated ulna fractures. Evaluating radiographs for plastic deformities of the ulna is crucial, as these injuries are often overlooked. Failed closed reductions may rise suspicion of interposed structures.

Isolated post-traumatic radial head dislocation is a rare occurrence requiring prompt recognition and management. While most cases are treated with closed reduction, those that are not reducible or suspected soft tissue interposition may require open reduction. Thorough clinical evaluation, both vascular and neurologic and preoperative imaging are essential. Early intervention and meticulous surgical techniques can lead to favorable functional outcomes.

## Introduction

The annular ligament of the elbow is crucial for elbow stability, playing a vital role in both the proximal radioulnar and humeroradial joints, as well as supporting the surrounding muscles and ligaments [1].

In children, the radial head is much more commonly subluxated than it is dislocated. Radial head subluxation, commonly referred to as nursemaid's elbow, is seen especially in children from six months to six years and often results from a pulling injury to the affected arm [2].

On the other hand, radial head dislocation is rare in children under eight years old, with the highest incidence of elbow dislocations occurring at 12-13 years of age [3,4]. This injury is often associated with ulna fractures, such as Monteggia injuries or plastic deformation injuries referred to as Monteggia variants [3,4]. In certain cases, of isolated radial head dislocation, the elbow cannot be reduced using closed reduction techniques, requiring surgical intervention. Possible reasons for this include delayed treatment, the presence of interposed tissues obstructing reduction, "buttonholing" of the radial head through the joint capsule, and severe elbow instability [4-6].

We herein present a case of an irreducible isolated radial head dislocation due to annular ligament interposition into the radiocapitellar joint in an 11-year-old male patient. This case highlights the diagnostic and management challenges associated with this rare condition and emphasizes the importance of recognizing annular ligament interposition as a potential barrier to closed reduction. By discussing our approach, we aim to contribute to the understanding of optimal treatment strategies for similar cases.

## Case presentation

**Figure 1:**
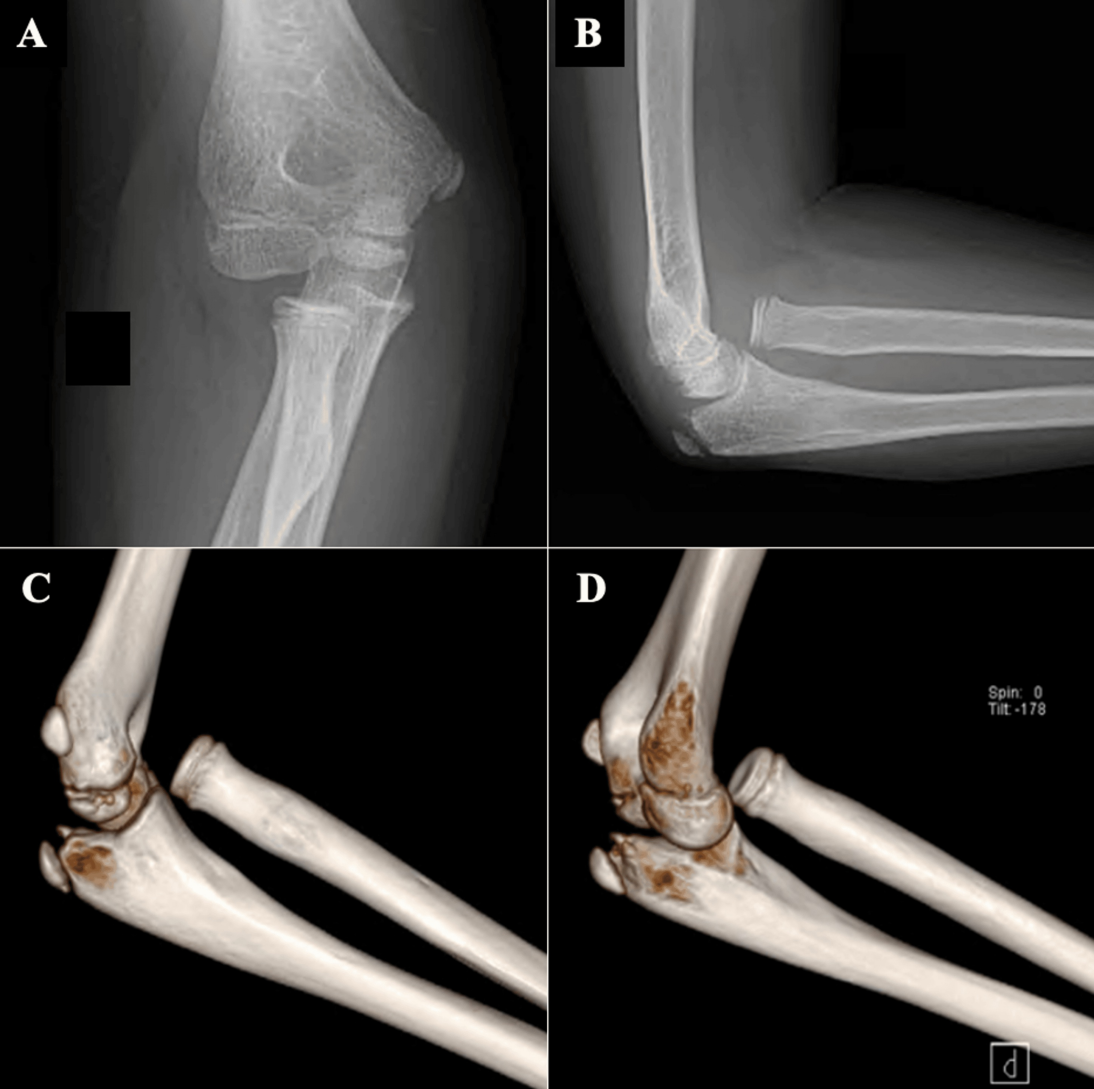
X-ray and computed tomography scan of radial head dislocation of the right elbow. 1A shows an anteroposterior radiographic view of radial head dislocation of the right elbow; 1B shows a lateral radiographic view of radial head dislocation of the right elbow; 1C and 1D show a 3D reconstruction of a computed tomography scan of radial head dislocation of the right elbow.

An 11-year-old boy was referred to our hospital after a fall from a standing height, with the right elbow in extension and pronation, resulting in indirect trauma to the elbow. As relevant background information, two years earlier, he had been diagnosed with a fracture of the right distal radius, which was conservatively treated with immobilization in a cast, without residual deformity compared to the contralateral side. The clinical presentation of the elbow included swelling, pain, severe functional limitation, deficits in flexion of the first and second fingers, and numbness in the second and third fingers. In the emergency room, an X-ray and a computed tomography (CT) scan were performed which showed an anteromedial dislocation of the radial head, with no fractures present (Figure 1).

An initial attempt at closed reduction was performed while the patient was awake, but it was unsuccessful. Subsequently, another attempt was made under general anesthesia, which also failed. The patient exhibited a restricted passive range of motion in flexion-extension and pronation-supination.

**Figure 2:**
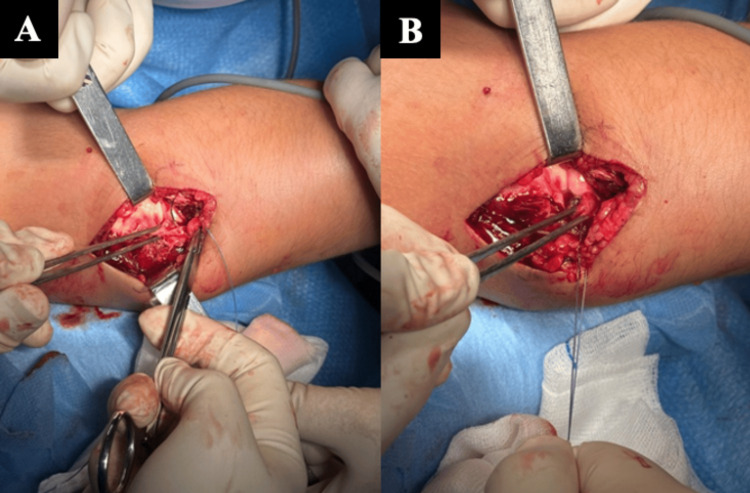
Identification and reattachment of the annular ligament. The images (A and B) illustrates the identification and reattachment of the annular ligament.

Since the closed reduction of the dislocation could not be achieved, surgery was opted for, using the Kaplan approach. The surgical exposure revealed an anterior dislocation of the radial head and interposition of the posterior half of the annular ligament which was infolded in the humeroradial space. The posterior half bundle of the annular ligament was removed from the posterior surface of the radial head, which could then be reduced and was primarily repaired with a 4-0 Vicryl suture to the anterior half still attached to the radial head (Figure 2).

**Figure 3:**
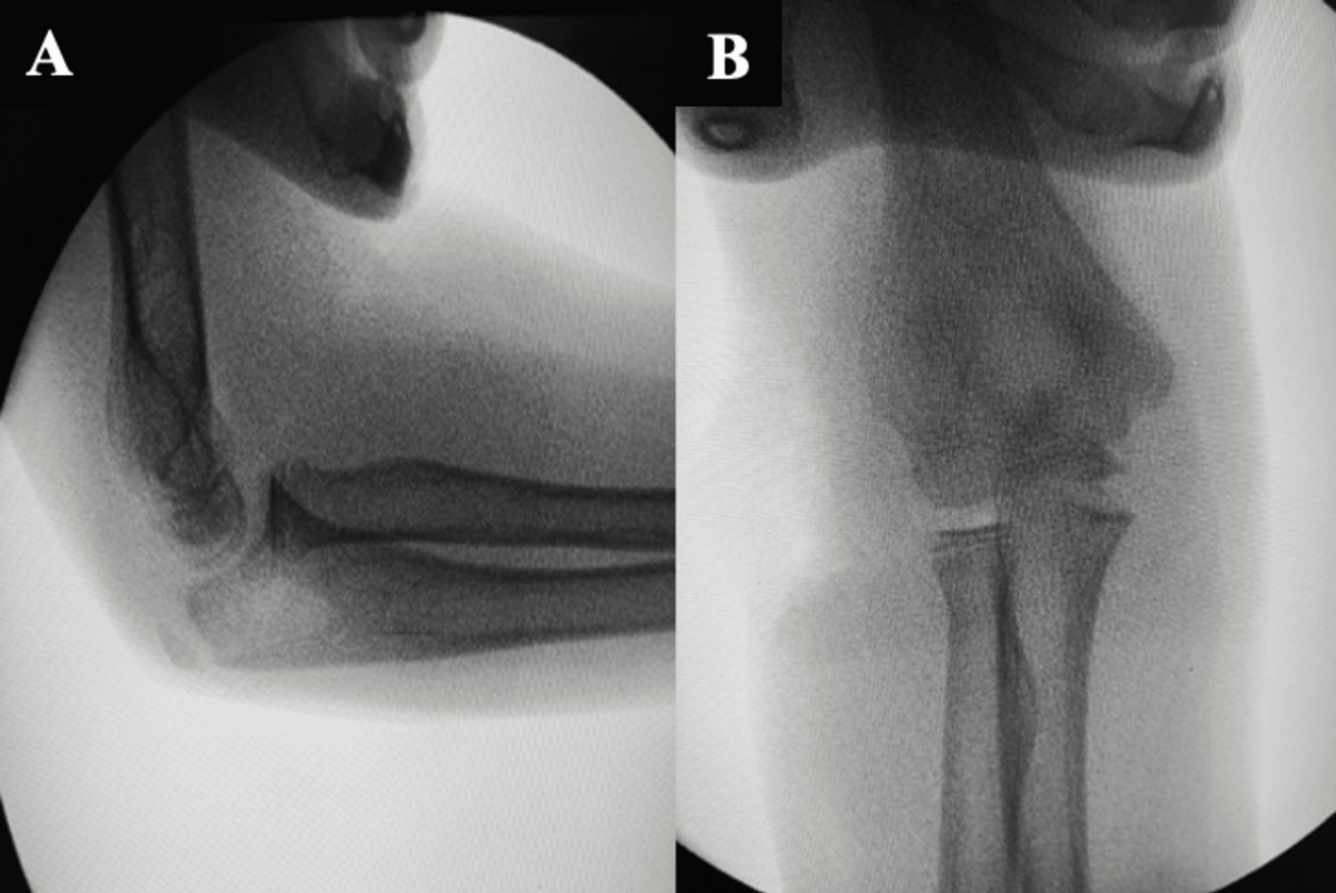
X-ray showing the intra-operative reduction of radial head dislocation of the right elbow. 3A shows the lateral radiographic view of the right elbow; 3B shows the anteroposterior radiographic view of the right elbow.

Concentric reduction was confirmed under fluoroscopy and a stable full range of motion was achieved (Figure 3).

A varus/valgus stress test was performed under fluoroscopy and was negative. Following the procedure, the patient's arm was immobilized in a plaster splint with the elbow flexed at 90° for one month.

In the immediate postoperative period, the patient exhibited a deficit in flexion of the first and second fingers (which we associated with anterior interosseous nerve neuropraxia). This deficit gradually decreased over the follow-up period.

**Figure 4:**
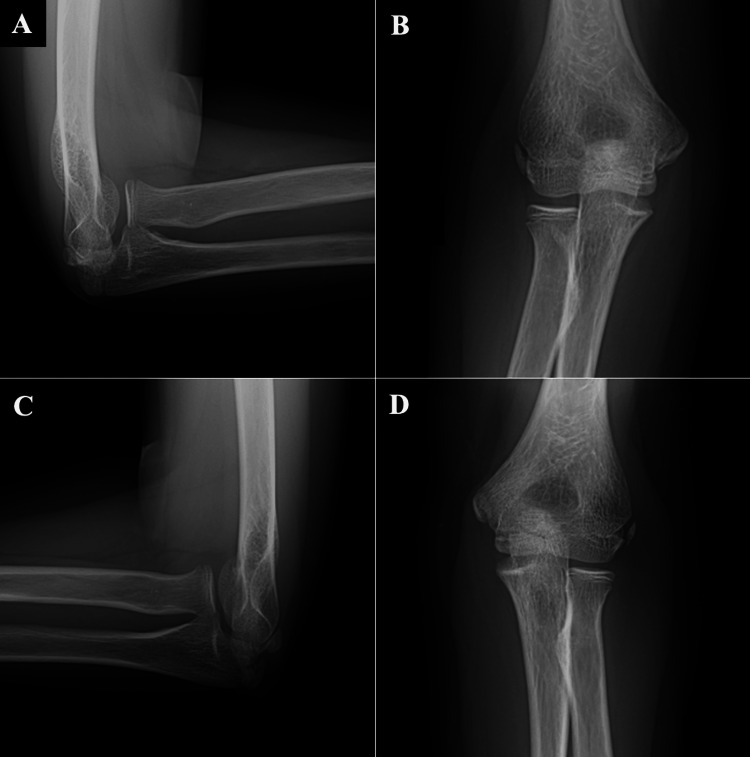
X-ray of right (A, B) and left (C, D) elbow at one year of follow-up. 4A shows a lateral radiographic view of the right elbow; 4B shows an anteroposterior radiographic view of the right elbow; 4C shows a lateral radiographic view of the left elbow; 4D shows an anteroposterior radiographic view of the left elbow.

Subsequent X-rays at two weeks, one month, six weeks, two months, three months, four months, six months, and 12 months (Figure 4) showed proper alignment of the radius and capitellum.

**Figure 5:**
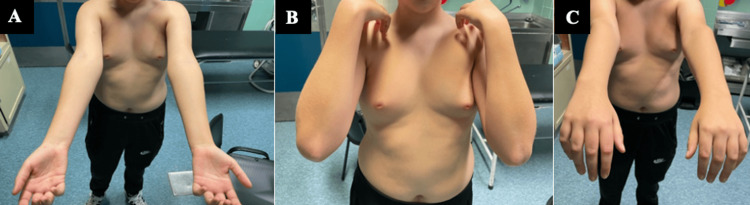
Symmetric flexion-extension and pronation-supination compared to the contralateral elbow. 5A shows symmetric supination and extension of the elbows; 5B shows symmetric flexion of the elbows; 5C shows symmetric pronation and extension of the elbows.

After one year of follow-up, the patient demonstrates a full range of motion of the elbow, with symmetric flexion-extension and pronation-supination compared to the contralateral elbow (Figure 5).

The patient now has a full range of motion in the first and second fingers and no longer experiences any paresthesia. The patient achieved a score of 7.76 on the Disabilities of the Arm, Shoulder, and Hand (DASH) scale and a score of 77 on the Upper Extremity Functional Index (UEFI). Regarding the Weber two-point discrimination test, the patient demonstrated normal results, being able to distinguish two points separated by 2 mm at the fingertip.

## Discussion

Radial head dislocation is uncommon in children, and when occurring without associated ulna fractures such as Monteggia injuries or plastic deformation injuries, it is even rarer [3,4]. It is crucial to thoroughly evaluate radiographs after a radial head dislocation in children to rule out a plastic deformity of the ulna, as this injury is often overlooked [6]. Normally, it can be treated with closed reduction, but when this does not occur, we have to think about other injuries such as delayed treatment, the presence of interposed tissues obstructing reduction, "buttonholing" of the radial head through the joint capsule, and severe elbow instability [4-6]. In our case, the patient did not present any injury on the X-ray other than radial head dislocation, but upon surgical exploration, we found an interposition of the annular ligament between the radial head and the capitellum, hindering the reduction of the radial head. However, without prompt treatment, more complex surgery would be required, as neglected radial head dislocations can lead to the annular ligament becoming trapped within the joint and subsequently sclerosed or necrotic under compressive load [7].

Acute traumatic radial head dislocations often result from direct elbow injury, a rare event where disruption of the annular ligament and anterior capsule is necessary for anterior dislocation to occur [8]. Several mechanisms for the onset of isolated radial head dislocation have been proposed. However, the specific injury mechanism responsible for isolated radial head dislocation remains a topic of debate [8,9]. For children with isolated traumatic radial head dislocation and annular ligament injury, surgery involving open reduction and annular ligament reconstruction is recommended [9]. Experts advocate this approach for all cases requiring open surgery on the radio-capitellar joint due to its importance in maintaining radial head stability [9].

It is important to note that in this case, the patient exhibited neurological deficits, being unable to flex the first and second fingers of the hand, and experiencing numbness in the second and third fingers. This was likely due to traumatic injury with the radial head impacting the median nerve. With ongoing physiotherapy, the patient fully recovered and currently has no motor or sensory deficits.

After one year, the patient has not experienced any new episodes of elbow dislocation and does not show any elbow instability.

## Conclusions

In conclusion, isolated post-traumatic radial head dislocation is a rare occurrence where prompt recognition and appropriate management are crucial. While most cases can be successfully treated with closed reduction, those presenting with anteromedial irreducibility or suspected soft tissue interposition may require open reduction for optimal anatomical restoration of the elbow joint. The consideration of capsular interposition as a potential barrier to reduction, especially in cases with minimal or correct ulnar alignment, underscores the importance of thorough clinical evaluation and when indicated, preoperative magnetic resonance imaging assessment. Despite the challenges posed by these injuries, early intervention and meticulous surgical techniques can lead to excellent functional outcomes.
